# Development and psychometric evaluation of a reflection on clinical practice questionnaire for nursing students

**DOI:** 10.1186/s12912-021-00705-0

**Published:** 2021-09-30

**Authors:** Mostafa Bijani, Fateme Mohammadi, Fariba Haghani, Nikoo Yamani, Shahnaz Karimi

**Affiliations:** 1 Department of Medical Surgical Nursing, Fasa University of Medical Sciences, Fasa, Iran; 2 Chronic Diseases (Home Care) Research Center and Autism Spectrum Disorders Research Center, Department of Nursing, Hamadan University of Medical Sciences, Hamadan, Iran; 3 Department of Medical Education, Medical Education Research Center, Isfahan University of Medical Sciences, Isfahan, Iran; 4 Department of Medical Education, Medical Education Research Center, Fasa University of Medical Sciences, Fasa, Iran

**Keywords:** Clinical practice, Nursing student, Psychometric, Nursing evaluation research

## Abstract

**Background:**

Reflection on clinical practice is the core of education for nursing students. Evaluating reflection on clinical experiences requires a tool which accurately measures reflection skills. The present study aims to develop and test the psychometric properties of a tool for measuring nursing students’ reflection on clinical practice.

**Methods:**

Based on a mix-method exploratory approach, the study was carried out in two stages: in the first stage (the qualitative phase), the concept of reflection on clinical practice was established. In the second stage (the quantitative phase), the psychometric properties of the developed scale were evaluated.

**Results:**

Based on the results of the exploratory factor analysis and the verification process, the final version of the scale came to consist of 36 items and 6 dimensions. The dimensions were: professional competence, internal sources of motivation, challenging situational clinical setting, dynamic organizational atmosphere, reflection-based self-management, and dynamic professional growth. Overall, 6 factors accounted for 62.79% of the variances. The factor loadings of the items ranged between 0.62 and 0.94, all of which were significant. The total intraclass correlation (ICC) of the scale was found to be 0.94. Also, evaluation of the reliability of the scale as measured through internal homogeneity yielded a total Cronbach’s alpha of 0.90.

**Conclusion:**

The findings show that the developed scale for evaluation of nursing students’ reflection on clinical practice possesses satisfactory validity and reliability, and nursing professors can use this instrument to assess students’ reflection skills.

## Background

Today, reflection on clinical practice is regarded as an integral part of education required for nursing students [1]. Graduates in nursing profession must provide healthcare in complicated, ever-changing environments [2]. In clinical environments, students experience various clinical challenges, for many of which there are not any definite solutions. Under these circumstances, reflection and reflective practice are stressed as essential parts of learning and education which enable learners to understand experiences and concepts and prepare them for coping with clinical challenges so that they can perform satisfactorily in new situations [3, 4].

The concept of reflection dates back to Dewey’s theory (1933). According to Dewey, learning comprises of experiencing combined with reflection on experiences. He believed that reflection provides for the intellectual activities required for dealing with professional challenges [5]. In fact, reflection means purposeful consideration of an experience towards learning, achieving a new insight, and improving one’s performance [6]. According to Boud, and Walker, reflection is a purposeful and conscious activity in which emotions and cognition are closely connected. They believe that learners who have a positive self-image are more likely to engage in reflective behaviors than those who have a negative self-image [7]. Mezirow believes that every adult has experiences by which he/she is surrounded. According to Mezirow, learners can achieve growth and development only if they can examine, evaluate and reflect on their preconceptions. Consequently, the learners’ preconceptions and accepted frameworks expand and may lead to beliefs and perspectives which better guide their behavior [8]. Researchers have attributed a variety of characteristics to reflection: development of practice-based knowledge, recognition of experience, correction and improvement in clinical practices, bridging the gap between theory and practice and, finally, development of a new learning tool [9]. Generally, reflection on experiences is a purposeful and skilled activity in which an individual exercises analysis and judgment to find a better substitute for action. Therefore, at the time of performing tasks or afterward, individuals should reflect on their activities and consider the related knowledge as well to reduce the gap between theory and practice and make learning through experience possible [10]. In a longitudinal study entitled “The Effect of a Self-Reflection and Insight Program on the Nursing Competence of Nursing Students,” Pai (2015) concludes that reflection on experiences in response to complexities in clinical practice reduces nursing students’ tension and improves their learning [11]. Mahlanze et al. (2016) reporte that reflective approaches encourage learners to seek more knowledge to be prepared for benefiting from new experiences and reduce the gap between knowledge and practice for them [12]. McGlinn states that reflection on experiences plays a significant role in improving surgeons’ ability to face similar experiences in the future and increases patients’ safety [13]. According to Schon (1987), reflection is formed in two steps: the first step to arrive at reflection is the existence of an issue—Dewey refers to this as awareness of a problem. To define this step, Schon uses the term “reflection-on-action.” The second step, called “reflection-in-action,” is going back to the issue for reconsideration [14]. “Reflective practice” is beyond “thoughtful practice.” Reflective practice is an in-depth understanding of experiences which helps individuals improved their behavior or performance [15]. Reflective practice is a key topic of increasing interest regularly raised in nursing-related fields and nursing educational programs. This kind of practice is essential to providing comprehensive, ethical, and safe nursing [16]. Also, as a learning tool, reflective practice can be used in nursing educational programs to improve critical thinking skills and self-guidance, plug the gap between theory and practice, and develop professional competencies [17]. In, nursing profession reflection can, by reducing the frequency of unconscious, habitual acts, enhance the quality of patient care and increase patient safety [18].

The purpose of training courses in nursing profession is to equip graduates’ with the necessary reflection skills; however, there is an obvious need for an instrument which measures learners’ ability to reflect on clinical experiences [19]. Learning and education should be designed so as to prepare learners to apply theoretical knowledge in real-life situations; and the real world is full of issues without predetermined answers. Furthermore, the real challenge of education is to prepare learners for the situations which they will face in their professional lives. Reflection can, as a learning strategy, prove very helpful in accomplishing the above-mentioned goals [20, 21].

A purposeful study of reflection on clinical practice requires an instrument which makes accurate assessment of reflection skills possible. Even though the goal of training courses in nursing profession is to improve graduates’ reflection skills, there is an obvious lack of an appropriate tool which measures preparation for reflection on clinical experiences [22].

Despite the fact that many reflective approaches are employed to enhance reflection on experiences in nurse education, the number of practical instruments for evaluating reflection on clinical experiences is very small. The first attempts at evaluating reflective thinking were made by Magolda (1987) who used qualitative methods and interviews with students to develop an instrument for measuring epistemological reflection [23]. Using Dewey’s views, Mezirow (1991) offered a theoretical framework for the different levels of reflective thinking and tried to differentiate between reflective action and non-reflective action. He suggested three types of non-reflective action, namely habitual action, thoughtful action and introspection. According to Mezirow, reflective action consists of two subcategories—process of reflection and content of reflection—which are lower than critical thinking. The dimensions suggested by Mezirow are as follows: habitual action, understanding or thoughtful action, reflection, and critical thinking [24].

Kamber (1996) explored reflective thinking by interviewing students and observing them in class. Kamber’s work has been compared with Mezirow’s classification of reflective thinking. The problem with Kamber’s work was that he only conducted periodic examinations of reflective writing and did not consider the periods before and after them to determine possible changes in the learners’ reflective thinking [25]. Kamber et al. (2000) claimed that there were few practical instruments for determining whether or not students exercised reflective thinking and, if they did, to what extent. Thus, they developed a brief questionnaire to evaluate the different levels of reflective thinking suggested by Mezirow in undergraduate students [26]. Kamber et al. (2000) addressed the four concepts of habitual action, recognition, reflection and critical reflection in Mezirow’s theory in their reflective thinking questionnaire. Kamber’s questionnaire is more suited for evaluation of reflective thinking in theoretical education in the classroom and measuring learners’ level of reflection or non-reflection [26].

The real world is filled with problems without predetermined solutions. Accordingly, the aim of learning and education, especially in nursing, should be to prepare individuals for using their theoretical knowledge in real life. The major challenge in clinical education is preparing learners for situations which they encounter in clinical learning environments. The goal of nurse education is to help nursing students learn from their experiences and acquire the necessary competence in giving comprehensive nursing care. Currently, nurse education is plagued by a gap between theory and practice. The best way to reduce this gap is to use reflective approaches. Reflection on experiences is a key component of clinical education in nursing. Despite the many training courses whose aim is to reduce the gap between theory and practice by encouraging reflection on experiences in clinical learning environments, evaluation of nursing students’ reflection on their clinical experiences has received very little attention. Moreover, a systematic and purposeful study of reflection on clinical experiences in nursing students is not possible without an instrument which allows for a comprehensive and accurate evaluation of that reflection. Studies show that despite the significance and practicality of Kamber’s reflective thinking scale, there is an urgent need for an instrument which comprehensively evaluates reflection on clinical experiences. Thus, the present questionnaire was developed to assess nursing students’ reflection on their experiences in clinical learning environments.

## Methods

The present study uses an exploratory-sequential mixed method design to develop and evaluate the psychometric properties of a questionnaire for measuring reflection on clinical practice in nursing students who are doing their bachelor’s degree studies in the south of Iran. According to Creswell (2014), one of the main applications of mixed method research is in instrument development. In order to develop an instrument which measures a healthcare-related concept, researchers must carefully identify and address the various aspects of that concept. Thus, it is essential that the component parts of the concept under study be examined carefully through appropriate methods and its various dimensions be determined clearly before an instrument for measuring the concept can be developed [27].

In the present study, to understand the process of reflection on clinical practice as applied by nursing students, the researchers used qualitative research with a grounded theory approach. Subsequently, in the second phase, the data collected from the first phase were used to develop and test an instrument.

### Ethical considerations

All participants gave written informed consent to participate in the study. The present study was conducted in accordance with the principles of the revised Declaration of Helsinki, a statement of ethical principles which directs physicians and other participants in medical research involving human subjects. Moreover, the study was approved by the local Ethics Committee of Fasa University of Medical Sciences, Fasa, Iran (IR.FUMS.REC.1397.178).

### Qualitative phase

#### Data collection

The qualitative phase study was conducted from Januuary 2018 to December 2019 in one of thesoutheastern cities of Iran. In the first phase, dimensions of the concept of Reflection on Clinical Practice were developed. In this regard, the researchers conducted semi-structured, in-depth interviews with 27 of the participants, consisting of 23 students, 2 instructors, and 2 nurse administrators. Sampling began with the selection of nursing students on a purposeful basis which was gradually, as codes and categories were extracted, replaced with theoretical sampling until data saturation was reached. The inclusion criteria were having passed at least one term of training in clinical environments, being willing to participate in the study, and being in physically and emotionally healthy conditions. The main approach to data collection was unstructured, in-depth interviews. Immediately after completion, each interview was transcribed verbatim. Then the transcripts were read and re-read several times. The researchers coded the transcripts to achieve an accurate understanding of the participants’ experiences. Codes were assigned in MAXQDA software.

##### Data analysis

In the present study, data were collected and analyzed simultaneously: after the first interview, data analysis began as well. The collected data were analyzed according to Strauss and Corbin’s method (1998) in three stages: open coding, axial coding, and selective coding [28]. This process of data analysis consisted of the following steps: careful reading of the transcripts and extraction of meaning units and main phrases, extraction of codes, classification of codes, establishment of sub-categories, identifying links between codes, determining the main variable, and emergence of the process of reflection on clinical practice.

The validity and reliability of the data were tested through the participants’ examination of the codes, peer review, and prolonged engagement with the subject of the study. The researchers engaged with the subject for over a year. For the participants to examine the data, parts of the transcripts with the initial codes were shown to the participants who were asked to compare the extracted codes with their responses. For peer review, the researchers submitted the concepts and categories extracted from the data to some fellow researchers familiar with qualitative research and had them examine and verify the relationship between the codes and the original data. Maximum variation sampling was applied to increase the transferability and credibility of the findings.

Analyses of the data yielded 6 main categories which represented nursing students’ manner of reflection on clinical practice: professional competence, internal sources of motivation, challenging situational clinical setting, dynamic organizational atmosphere, reflection-based self-management, and dynamic professional growth.

##### Item generation

At the end of the qualitative stage, the items of the scale were developed: in line with the qualitative objective, a definition of the concept of reflection on clinical practice and its constituent dimensions were established. Next, using the blueprint, the researchers generated a pool of questions on the categories and subcategories included in the definition of reflection on clinical practice. Then the items were determined according to the qualitative findings of the study (inductive approach). The items were completed using a review of literature and similar questionnaires (deductive approach). The initial draft of the reflection on clinical practice scale consisted of 75 items, which, after a review of literature, increased to 85 items.

### Quantitative phase

In the second stage of the study, the psychometric properties of the instrument were tested. The face validity of the scale was measured both qualitatively and quantitatively (item impact coefficient). Likewise, the content validity of the scale was measured both qualitatively and quantitatively (SCVI/Ave, S-CVI, CVI, and CVR). The researchers also carried out an item analysis (Cronbach’s alpha) and tested the construct validity (via confirmatory and exploratory factor analyses) and reliability (internal consistency and test-retest reliability) of the new scale.

#### Face validity

To determine the face validity of the scale qualitatively, the researchers interviewed 10 undergraduate nursing students, 3 nursing professors, and 2 literary editors about the difficulty level, clarity, and grammatical accuracy of the items. The information thus collected resulted in the revision of the some of the items, but none of the items was eliminated. After the items had been revised, the quantitative evaluation of the face validity of the scale was performed using the item impact score method. Accordingly, 10 members of the target group (undergraduate nursing students) were asked to rate the significance of each item on a 5-point Likert scale (5 = very important, 4 = important, 3 = relatively important, 2 = not very important, 1 = not important at all). Subsequently, the impact score of each item was calculated. Impact scores of over 1.5 were considered to be satisfactory [29].

##### Content validity

The qualitative measurement of the content validity of the scale was carried out based on the opinions of 15 nursing experts with adequate knowledge and experience in the field of instrument development and with experience of clinical practice. At this point, the grammatical accuracy, appropriateness of the terms, necessity of the items, placement of the items, and scoring were assessed. The researchers executed the quantitative evaluation of the content validity of the scale by calculating the content validity ratio (CVR) of the items (to determine the necessity of the items) and the content validity index (CVI) of the items (to determine the relevance of the items to the concept of reflection on clinical practice). In addition, the researchers calculated the Kappa coefficient to determine inter-rater agreement without considering the probability of chance agreement and the total content validity index (S-CVI) of the instrument. The initial draft designed on a 3-point Likert scale (necessary, helpful but not necessary, and unnecessary) was evaluated by 15 experts based on whose views the content validity ratio (CVR) of the scale was determined. According to Lawshe’s table, items whose numerical values are equal to or above 0.49 are kept [30]. CVI was measured based on Waltz and Bausell’s criteria. Accordingly, the relevancy, clarity, and simplicity of each item were rated by 15 experts on a 4-point Likert scale. Numerical values of more than 0.78 were considered to be satisfactory [31]. The researchers also calculated the Kappa statistic to measure inter-rater agreement without considering the probability of chance agreement—Kappa coefficients of between 40 and 59% are regarded as “relatively satisfactory” and between 60 and 74% are regarded as “excellent”. The total content validity (S-CVI) of the instrument was measured by calculating its average content validity index (SCVI/Ave). According to Polit and Beck (2016), average content validity indexes of more than 0.90 are satisfactory [32].

##### Construct validity

Sample size.

In the present study, construct validity was measured via exploratory factor analysis. Many studies suggest a sample size of 5 to 10 subjects per item for factor analysis [33]. In this study, the number of sampled subjects was 10 times the number of the items on the scale, i.e. 360 nursing students.

Exploratory factor analysis.

Exploratory factor analysis was executed through Kaiser-Meyer-Olkin (KMO) sampling adequacy test, Bartlett’s test of sphericity, analysis of the main factors, a scree plot, and varimax rotation with a sample size of 360 subjects. In the first stage of factor analysis, sampling adequacy was tested using KMO’s test. The KMO index varies between 0 and 1, with higher values indicating better suited data for factor analysis. Values of more than 0.9 are considered to be excellent, and values of more than 0.8 are regarded as satisfactory [23, 34]. To determine whether factor analysis was justified based on the correlation matrix, in other words, whether there was enough correlation among the items to merge them, the researchers employed Bartlett’s test of sphericity. After the calculation of the correlation matrix among the items, factors were extracted. The factor loading of each item in the factor matrix and rotated matrix must be at least 0.4 [35]. In the present study, a factor loading of 0.4 was taken as the least acceptable degree of correlation between each item and the extracted factors.

### Reliability

The reliability of the instrument was tested by measuring its internal consistency (Cronbach’s alpha coefficient) and test-retest reliability. Internal consistency was measured with a sample of 360 nursing students. A Cronbach’s alpha of 0.7–0.8 proved the internal consistency of the scale to be satisfactory [36]. The consistency of the instrument was tested via the test-retest method: 100 nursing students completed the scale twice with a two-week interval. Then the two sets of test scores were analyzed using the intraclass correlation coefficient for each of the subscales and the entire scale. Grimani (2017) recommend a two-week to one-month interval for testing the consistency of a questionnaire. An index of more than 0.8 is proof of satisfactory consistency of an instrument [37].

## Results

In the qualitative phase of the study, the concept of reflection on clinical practice was found to consist of the following 6 domains: professional competence, internal sources of motivation, challenging situational clinical setting, dynamic organizational atmosphere, reflection-based self-management, and dynamic professional growth. Developed based on the findings of the qualitative phase, the initial draft of the reflection on clinical practice scale consisted of 75 items, which, after a review of literature, increased to 85 items. Some of the items which were found to be similar in meaning by the research team were merged, thereby reducing the number of the items to 65. The face validity of the scale was evaluated qualitatively using the index of item impact score. Except for 15 items, all the items obtained an impact score of more than 1.5. Thus, the number of the items shrank from 65 to 50. Evaluation of the content validity ratio (CVR) of the items showed that 8 items had a score of less than 0.49 and were, therefore, eliminated. Thus, the scale entered the content validity index measurement phase with 42 items. Considering the cut-off point of 0.78 for the content validity index in the present study, the index value of all the items, except for 6 items, was over the possible minimum. Accordingly, the number of items reduced to 36. The Kappa coefficient of all the remaining 36 items was found to be 0.89, which is considered to be excellent. Also, the SCVI/Ave. of the scale was calculated to be 0.91, which is considered to be very high. To measure the construct validity of the scale, the researchers initially calculated the sampling adequacy index using the KMO test. The index was found to be 0.921. The result of Bartlett’s test of sphericity showed that chi-square with the value of 10,750.409 and degree of freedom of 630 was significant at *p* < 0.0001. These findings confirmed the results of the KMO test.

**Fig. 1:**
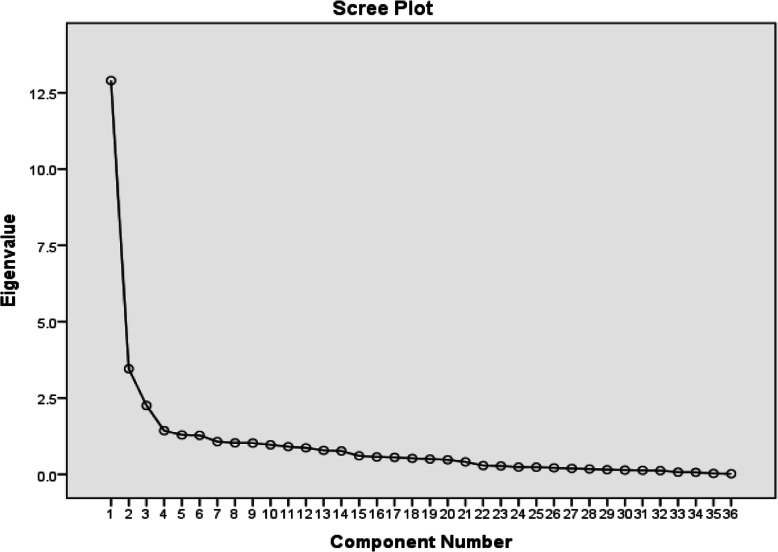
The factor analysis scree plot

Based on the scree plot, 6 factors were found to be dominant in the scale (Fig. 1). The results of the exploratory factor analysis showed that the 6 factors overall determined 62.79% of the variance. The factor loadings of the variables ranged between 0.60 and 0.94 and were all significant (Table1). The 6 domains introduced in the main scale were verified with acceptable values. The domain of professional competence consisted of 10 items (items 1–10), internal sources of motivation consisted of 5 items (items 11–15), challenging situational clinical setting consisted of 5 items (items 16–20), dynamic organizational atmosphere consisted of 5 items (items 21–25), reflection-based self-management consisted of 6 items (items 26–31), and dynamic professional growth consisted of 5 items (32–36). (Table1).

**Table 1:** Factor structure and factor load of each item using a varimax rotation

Factors’ names	Item	Factor loading
Factor 1: Professional competence	Q1. I am willing to perform specialized, complex, non-routine activities.	0.80
	Q2. I perform my duties as a nurse with care and concentration	0.94
	Q3. I try to keep my academic knowledge and clinical skills up-to-date	0.73
	Q4. I perform my duties with self-confidence	0.89
	Q5. I treat clinical situations with curiosity	0.85
	Q6. I am willing to acquire independence in performing my nursing duties	0.92
	Q7. I am not afraid of encountering difficult clinical situations and performing complex procedures	0.86
	Q8. I imagine myself in my patients’ situations in order to understand and analyze their problems better	0.87
	Q9. I feel responsible for solving my patients’ problems and relieving their pain	0.83
	Q10. I am accountable for my activities as a nurse	0.80
Factor 2: Internal sources of motivation	Q11. My beliefs help me reflect on my clinical practice	0.75
	Q12. My work conscience helps me reflect on my clinical practice	0.73
	Q13. My belief in showing respect for human values helps me reflect on my clinical practice	0.72
	Q14. My interest in the field of nursing helps me reflect on my clinical practice	0.70
	Q15. My desire for progress and success helps me reflect on my clinical practice.	0.69
Factor3: Challenging situational clinical setting	Q16. Encountering complicated clinical situations helps me reflect on my clinical practice	0.67
	Q17. Encountering questions and challenges in the clinical environment helps me reflect on my clinical practice.	0.73
	Q18. Fear of making mistakes in clinical situations helps me reflect on my clinical practice.	0.75
	Q19. Inadequate academic and practical preparation for performing clinical activities makes me reflect on my clinical practice.	0.68
	Q20. Clinical behaviors contrary to the principles of patient care make me reflect on clinical practice.	0.78
Factor 4: Dynamic organizational atmosphere	Q21. My instructors’ feedback and assignments related to patient care make me reflect on encountered situations.	0.70
	Q22. Having to perform nursing activities makes me reflect on encountered situations or my performance as a nurse.	0.68
	Q23. Active learning methods, e.g. formulating questions and case-based learning, make me reflect on encountered situations or my performance as a nurse	0.67
	Q24. Interaction with my instructors makes me reflect on encountered situations or my performance as a nurse.	0.65
	Q25. Interprofessional relationships based on respect make me reflect on encountered situations or my performance as a nurse.	0.62
Factor5: Reflection-basedself-management	Q26. When reflecting on clinical situations, I reflect on the consequences of my measures as a nurse.	0.73
	Q27. When reflecting on clinical situations, I contemplate all the events in my mind.	0.68
	Q28. I analyze clinical matters from different perspectives in my mind	0.62
	Q29. When reflecting on clinical situations, I engage in self-	0.63
	questioning to find solutions to problems.	
	Q30. When reflecting on taken clinical measures, I consult others to obtain the information I need.	0.74
	Q31. When reflecting on taken clinical measures, I search in scientific sources to obtain the information I need.	0.75
Factor 6: Dynamic professional growth	Q32.Reflecting on clinical situations helps me do my job correctly and according to the principles of professional ethics	0.74
	Q33.Reflecting on clinical situations helps me provide evidence-based care.	0.73
	Q34.Reflecting on clinical situations helps me share my experiences with others to improve the quality of nursing care.	0.74
	Q35. Reflecting on clinical situations helps me feel calm and satisfied.	0.68
	Q36. Reflecting on clinical situations helps me participate in patient education	0.67

The reliability of the instrument**:** The internal consistency of the scale was determined by measuring its Cronbach’s alpha. The coefficient was calculated for all the factors (subscales) and the entire scale with a sample size of 360 subjects. Cronbach’s alphas of more than 0.7 are considered to be acceptable. The findings showed that the present scale had very good reliability with a Cronbach’s alpha of 0.90. The consistency of the scale was determined using the test-retest approach. The intraclass correlation coefficient (ICC) of the whole scale was found to be 0.94 and significant at *p* < 0.05 (Table 2). The final version of the evaluation of a reflection on clinical practice questionnaire for nursing students included 36 items. All the items on the final draft of the scale are scored positively on a 5-point Likert scale: Always = 5, Usually = 4, Occasionally = 3, Seldom = 2, and Never = 1 (Table 3).

**Table 2:** The scor and the intraclass correlation coefficient (ICC) values of the reflection on clinical Practice for nursing students

Factor	Dimensions	ICC	Confidence interval (0.95)	*P* -value
1	Professional competence	0.91	0.79–0.878	*p* < 0.05
2	Internal sources of motivation	0.90	0.87–0.95	*p* < 0.05
3	Challenging situational clinical setting	0.86	0.82–0.91	*p* < 0.05
4	Dynamic organizational atmosphere	0.89	0.79–0.94	*p* < 0.05
5	Reflection-based self-management	0.89	0.82–0.92	*p* < 0.05
6	Dynamic professional growth	0.90	0.85–0.94	*p* < 0.05
Total		0.94	0.88–0.97	*p* < 0.05

**Table 3:** The Reflection on Clinical Practice Questionnaire for Nursing Students (36 items)

Item	Always	Usually	Occasionally	Seldom	Never
1. I am willing to perform specialized, complex, non-routine activities.					
2. I perform my duties as a nurse with care and concentration.					
3. I try to keep my academic knowledge and clinical skills up-to-date.					
4. I perform my duties with self-confidence.					
5. I treat clinical situations with curiosity.					
6. I am willing to acquire independence in performing my nursing duties.					
7. I am not afraid of encountering difficult clinical situations and performing complex procedures.					
8. I imagine myself in my patients’ situations in order to understand and analyze their problems better.					
9. I feel responsible for solving my patients’ problems and relieving their pain.					
10. I am accountable for my activities as a nurse.					
11. My beliefs help me reflect on my clinical practice.					
12. My work conscience helps me reflect on my clinical practice.					
13. My belief in showing respect for human values helps me reflect on my clinical practice.					
14. My interest in the field of nursing helps me reflect on my clinical practice.					
15. My desire for progress and success helps me reflect on my clinical practice.					
16. Encountering complicated clinical situations helps me reflect on my clinical practice.					
17. Encountering questions and challenges in the clinical environment helps me reflect on my clinical practice.					
18. Fear of making mistakes in clinical situations helps me reflect on my clinical practice.					
19. Inadequate academic and practical preparation for performing clinical activities makes me reflect on my clinical practice.					
20. Clinical behaviors contrary to the principles of patient care make me reflect on clinical practice.					
21. My instructors’ feedback and assignments related to patient care make me reflect on encountered situations.					
22. Having to perform nursing activities makes me reflect on encountered situations or my performance as a nurse.					
23. Active learning methods, e.g. formulating questions and case-based learning, make me reflect on encountered situations or my performance as a nurse.					
24. Interaction with my instructors makes me reflect on encountered situations or my performance as a nurse.					
25. Interprofessional relationships based on respect make me reflect on encountered situations or my performance as a nurse.					
26. When reflecting on clinical situations, I reflect on the consequences of my measures as a nurse.					
27. When reflecting on clinical situations, I contemplate all the events in my mind.					
28. I analyze clinical matters from different perspectives in my mind.					
29. When reflecting on clinical situations, I engage in self-questioning to find solutions to problems.					
30. When reflecting on taken clinical measures, I consult others to obtain the information I need.					
31. When reflecting on taken clinical measures, I search in scientific sources to obtain the information I need.					
32. Reflecting on clinical situations helps me do my job correctly and according to the principles of professional ethics.					
33. Reflecting on clinical situations helps me provide evidence-based care.					
34. Reflecting on clinical situations helps me share my experiences with others to improve the quality of nursing care.					
35. Reflecting on clinical situations helps me feel calm and satisfied.					
36. Reflecting on clinical situations helps me participate in patient education.					

## Discussion

The present study was an attempt at developing and testing the psychometric properties of a scale for measuring reflection on clinical practice. The developed scale addresses a wide range of the factors which constitute reflection on clinical practice in 6 domains: professional competence, internal sources of motivation, challenging situational clinical setting, dynamic organizational atmosphere, reflection-based self-management, and dynamic professional growth. Evaluation of the psychometric properties of the scale proved it to be satisfactorily reliable and valid. A thorough review of literature showed that there were not any instruments specifically designed for assessment of reflection on clinical practice. Therefore, what follows is the outcome of a review of relatively similar studies in this area.

In 2019, Rogers et al. used a researcher-made questionnaire—Reflective Practice Questionnaire (RPQ)—to measure medical students’ reflection skills. RPQ is a self-report instrument consisting of 16 items in four domains: reflection-in-action, reflection-on action, reflection with others, and self-appraisal. The internal consistency of this instrument has been verified with a Cronbach’s alpha of 0.84. Even though some of the items of this questionnaire address reflection on clinical experiences, the other aspects of students’ reflection on clinical experiences are not dealt with. Moreover, the study does not provide any explanation about the manner of evaluation of the content validity and construct validity of the questionnaire. Also, the items of this instrument have been developed based on a review of literature only and students’ perceptions (qualitative approach) have not been taken into account. In the present study, however, the items of the scale were developed using both a qualitative approach and a review of literature and, thus, the scale measures a wider range of the aspects of nursing students’ reflection on clinical practice, which is one of the strengths of the present scale [38].

In their study, Chong et al. (2009) use a researcher-made questionnaire to evaluate nursing students’ perception of reflective practice. Their questionnaire consists of 37 items. The items of the questionnaire address three domains: the first domain concerns students’ perception (24 items), the second domain is related to reflective practice (7 items), and the third domain deals with the subjects which students reflect on (6 items). The items are scored on a 5-point Likert scale, ranging from “completely agree” = 5 to “completely disagree” = 1. Chong et al. conclude that students consider reflective practice to be useful.

The content validity of the above-mentioned questionnaire has been verified by 5 nursing professors. In the present study, however, the content validity of the developed scale was assessed by a panel of 15 experts. Also, Chong et al. have not tested the face validity and construct validity of their instrument. And the items of their questionnaire have been composed solely based on a review of literature, without any use of the qualitative approach. Even though Chong’s questionnaire includes a few items which address reflection on clinical experiences, it fails to comprehensively evaluate the various aspects of nursing students’ reflection on clinical practice. In the present study, however, the items were developed according to nursing students’ views and experiences, as well as an extensive review of academic literature (an inductive-comparative approach), and all the psychometric properties of the designed scale were fully evaluated, making the present scale a more appropriate tool for measuring the different domains of reflection on clinical experiences [39].

The reflective thinking questionnaire, developed by Kember et al. in 2000, is a researcher-made instrument which consists of 16 items. The items assess undergraduate students’ reflective thinking in 4 domains: habitual action, understanding, reflection, and critical reflection. The items of this questionnaire have been developed based on a review of literature only and students’ experiences have not been taken into consideration. The reliability of the instrument has been verified with a Cronbach’s alpha of 0.89, and the other psychometric properties of the scale have been tested completely (via exploratory and confirmatory factor analyses); yet, the reflective thinking questionnaire measures reflective thinking and is not fit for evaluating the domains of reflection on clinical experiences. In fact, Kember states that this questionnaire has been designed for use in academic environments and will need to be modified if it is to be used in clinical environments. The primary application of the reflective thinking questionnaire is in assessing the impact of learning environments and education on learners’ reflective thinking. This scale can be used at the beginning and end of academic courses to determine the effects of the courses on students’ reflective thinking. The results can help policy-makers improve the content and settings of education in order to enhance students’ reflective thinking skills [26].

### Strengths and limitations

The greatest strength of this study is that it developed a specific tool for assessing reflection on clinical practice for nursing students. Moreover, the tool was generated through both deductive and inductive methods. Deductive-inductive concept analysis is the right approach for assessing reflection on clinical practice for nursing students and developing assessment tools. One of our limitations is that, the psychometric properties of the questionnaire were conducted in only one city. Therefore, it is recommended that future studies are carried out in other cities and contexts. Also, confirmatory factor analysis and determination of cut-off points were not implemented, that are planned for future studies.

## Conclusion

The present scale is a valid and reliable instrument for evaluating reflection on clinical practice. Nursing professors can use this scale to measure nursing students’ reflection on clinical experiences in clinical environments and use the results to identify the weaknesses in educational programs and take the necessary steps to improve the students’ reflection skills. Such measures will increase the students’ professional competencies which will, in turn, increase the quality and effectiveness of clinical services provided to patients.

## Data Availability

The datasets used and/or analysed during the current study are available from the corresponding author on reasonable request.

## Abbreviations

CVR: Content validity ratio
CVI: Content VALIDITY INDEX
ICC: Intraclass correlation coefficient
